# Supplementary material for: Generation of severely scoliotic subject-specific musculoskeletal models
Source: PLoS One. 2025 Dec 1;20(12):e0336211. doi: 10.1371/journal.pone.0336211 (PMC12668498; doi:10.1371/journal.pone.0336211)
Supplement: S1 File — Protocol for performing the virtual palpation of the anatomical landmarks and the accompanying naming protocol for the landmarks. (PDF) [file pone.0336211.s001.pdf]

Protocol for the virtual palpitation of landmarks on the scoliotic spine

---

This document details the protocol for placing markers on a CT scan (with the 3D model from a very quick segmentation achieved with a single thresholding operation) of a scoliotic spine using either Slicer3D or Mimics. The markers will be used to generate an OpenSim model of the scoliotic spine from the healthy spine model of Bruno et al. 2015.

Please read the section on the virtual landmark palpitations positions and the naming convention carefully (starting on page 2).

If you are using Slicer3D the protocol starts on page 9.

If you are using Mimics the protocol starts on page 14.

You are welcome to do the virtual palpitation in any software you want only requirement is that the virtual landmarks are placed correctly, and that you can export the virtual landmarks with the names (naming convention needs to be followed) and coordinates.

Virtual Marker Palpitations Positions

This section first describes very briefly the anatomy of the spine and the sacrum. Then the naming convention of the markers is detailed and then finally the position and order in which to place them is explained.

Anatomy of spine/sacrum:

S1 Fig 1: Anatomy of a vertebra.

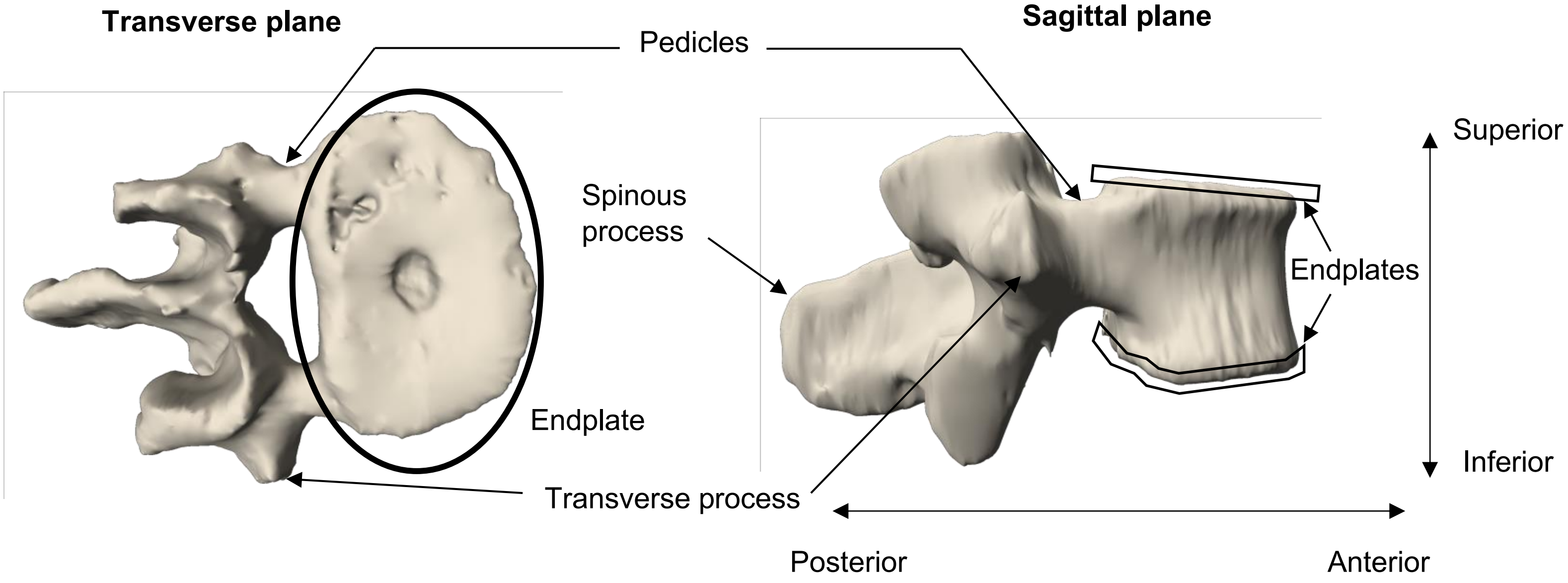

Pedicles connect the vertebral body to the processes. For the purpose of this virtual palpitation only the vertebral body and the pedicles are of interest.

On the next page is a picture of the spine showing how the vertebra and joints are labelled.

S1 Fig 2: Anatomy of the sacrum

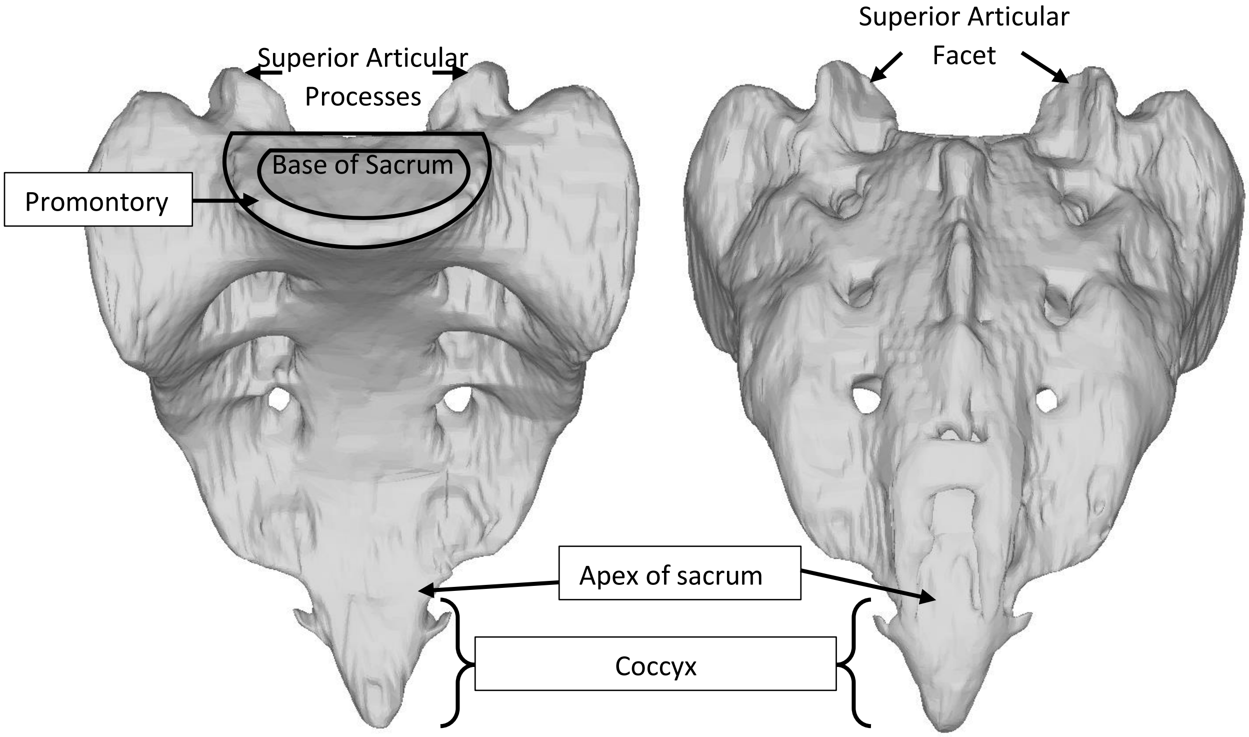

S1 Fig 3: Spine with vertebra level and joint levels labels.

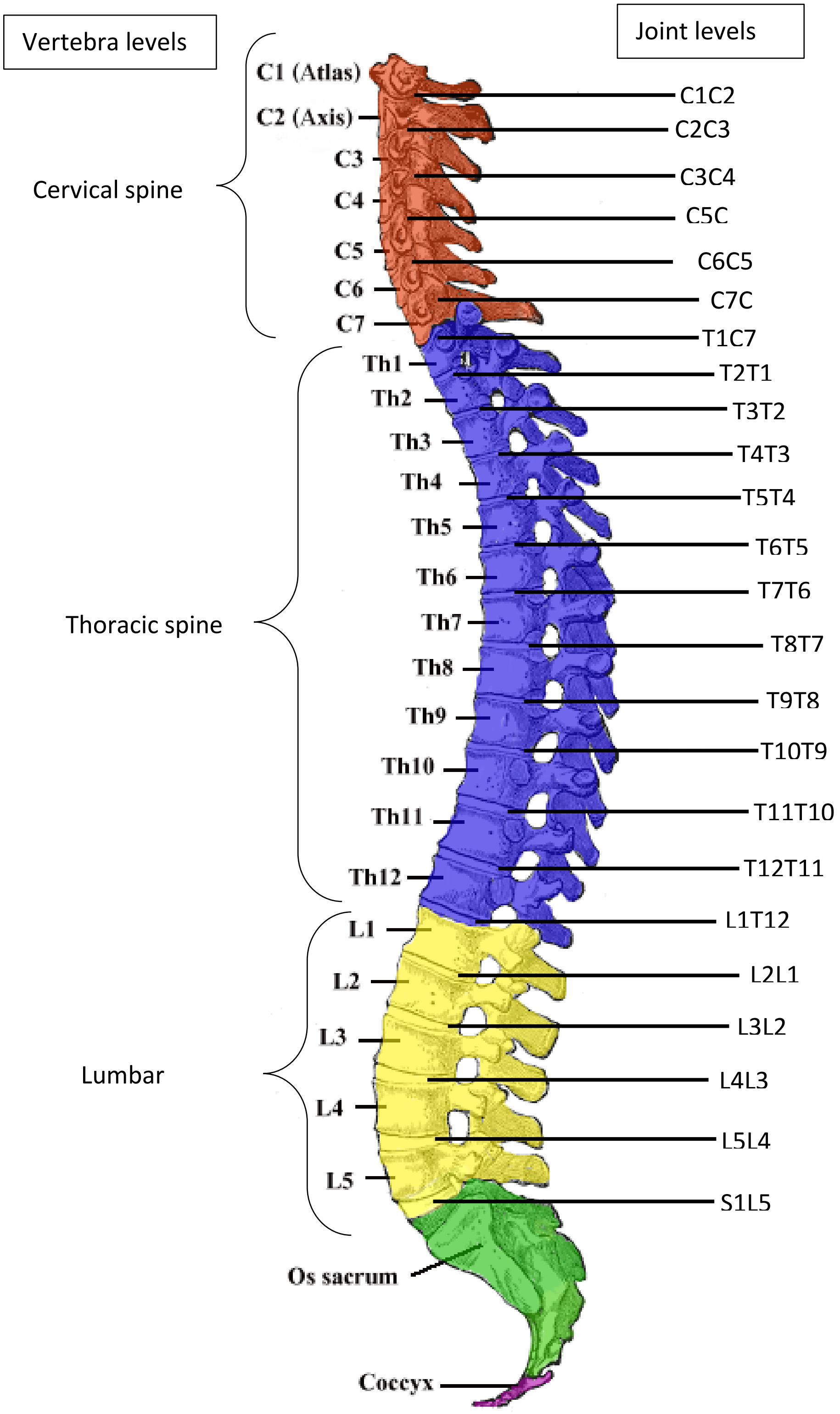

[This Photo] by Unknown Author is licensed under [CC BY-SA]. It has been modified to include the labels of the spinal regions and the joint levels

Summary of naming convention:

Abbriviations:
VI – inferior vertebra
VC – current vertebra
VS – superior verterba
JM – joint marker

A total of 14 markers are placed per vertebra and 8 markers on the sacrum. Naming convention needs to be followed.

Two virtual landmark catagories:

- Joint markers, 6 per vertebra
- Scaling markers, 8 per vertebra

They can be distiguished by the name. The names have two parts.

- Joint markers:
  - Naming has two parts
    - Marker labeled, JM#_ (# = number 1-6)
    - Joint level, VIVC or VCVS
    - Example: centre of endplate marker on the L5 vertebra for the joint between L5 and L4 would be JM1_L5L4. 1 corrisponds to the marker label ie. centre of the top endplate, L5 corrisponds the current vertebra ie. the vertebra the marker is being placed on and L4 the vertebra adjacent to the joint, so L5L4 corrisponds to VCVS. If instead we were looking at the marker for the centre of endplate on the L5 vertebra for the joint S1 and L5 then VIVC would be used (S1L5).
  - Markers 1-3 go on top endplate
  - Markers 4-6 go on bottom endplate
- Scaling markers
  - Name has two parts
    - Vertebra level, VC_
    - Marker label, M# (# = number 1-8)
    - Example: anterior most marker on the top endplate of lowermost lumbar vertebra would be L5_M1. Where L corrisponds to lumbar vertebra (for sacra it would be S, thoracic T, and cervical C), 5 to the lowermost lumbar vertebra, and 1 corrisponds to the marker label ie. the anterior most marker on the top endplate
  - Markers 1-4 go on top endplate
  - Markers 5-8 go on bottom endplate

Details on marker naming:

The logic of the marker numbering is that it starts at the most superior anterior marker and increases going around the endplate in the clockwise direction (looking at it in the superior→inferior direction) and then increases as you move inferior, which then again increases as you move round the endplate in

the clockwise direction (superior→inferior direction). Numbering restarts following the same logic when you move to a new vertebra.

S1 Fig 4: Locations of the virtually palpated anatomical landmarks on the sacrum and the labelling convention for the markers.

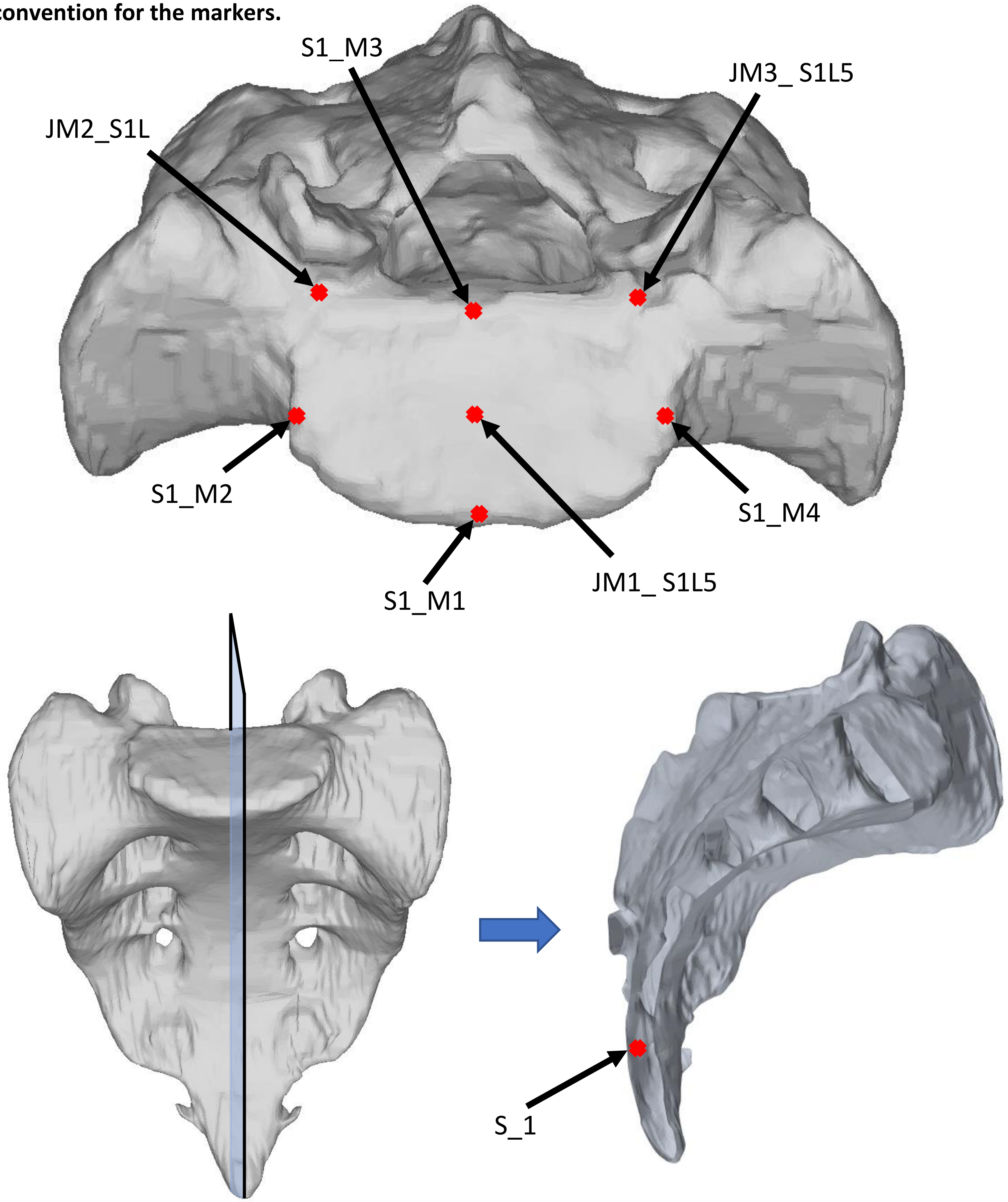

S1 Fig 5: Locations of the virtually palpated anatomical landmarks on the vertebrae and the labelling convention for the markers. VC = current vertebra level, VS = superior vertebra level, VI = inferior vertebra level.

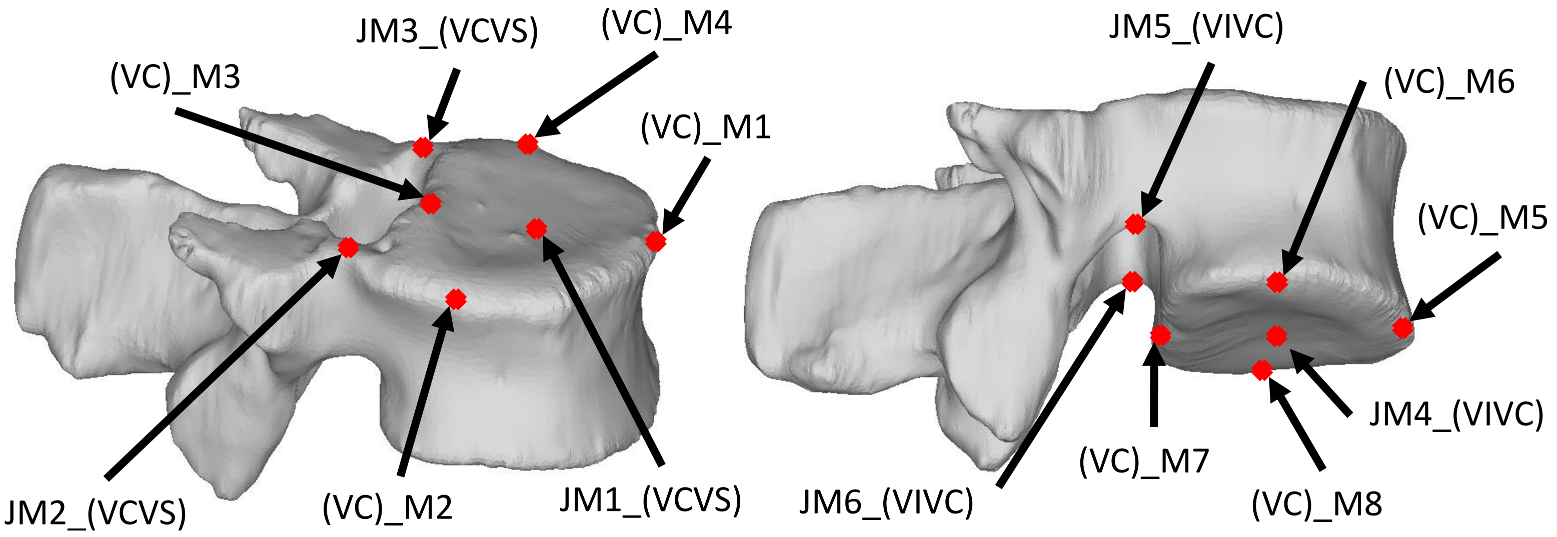

Marker placement:

Order of marker placement:

First marker to place is at the apex of the sacrum (S_1).

After that move upwards placing markers endplate by endplate.

I recommend placing the anterior and posterior markers first, followed by lateral markers, then the centre marker and finally the markers on the pedicles.

Sacrum Markers:

S_1 should be placed at the centre (right-left and anterior-posterior) of the apex of the sacrum.

M1 and M3 should be placed at the anterior and posterior most points of the promontory.

M2 and M4 should be placed at the anatomical right and left most points of the promontory.

JM1_S1L5 should be placed at the centre of the base of the sacrum

JM2 and JM3 should be placed at the lowermost point on the anatomical right and left superior, respectively, surfaces on the part connecting the promontory to the superior articular process. They should **NOT** be on the base/promontory of the sacrum.

Joint markers:

JM4 and JM1 should be placed in the centre of the endplates.

JM2 and JM3 on the inferior most part of the superior surface of the (anatomical) right and left pedicles respectively.

JM5 and JM6 on the superior most part of the inferior surfaces of the (anatomical) right and left pedicles respectively, **NOT** on the processes of the vertebra.

Scaling makers:

Markers M5 and M7 should be placed on the anterior and posterior most point of the inferior endplate respectively.

Markers M1 and M3 should be placed on the anterior and posterior most point of the superior endplate respectively.

- M1, M3, M5, and M7 should roughly align with the spinous process provided it is not deformed.

Markers M6 and M8 should be placed on the anatomical right and left sides on the vertebra inferior endplate respectively.

Markers M2 and M4 should be placed on the anatomical right and left sides on the vertebra inferior endplate respectively.

- M2, M4, M6, M8 should be approximately equidistant from the markers on the anterior and posterior of the vertebra.

For Slicer3D:

Interface:

S1 Fig 6: Slicer3D interface.

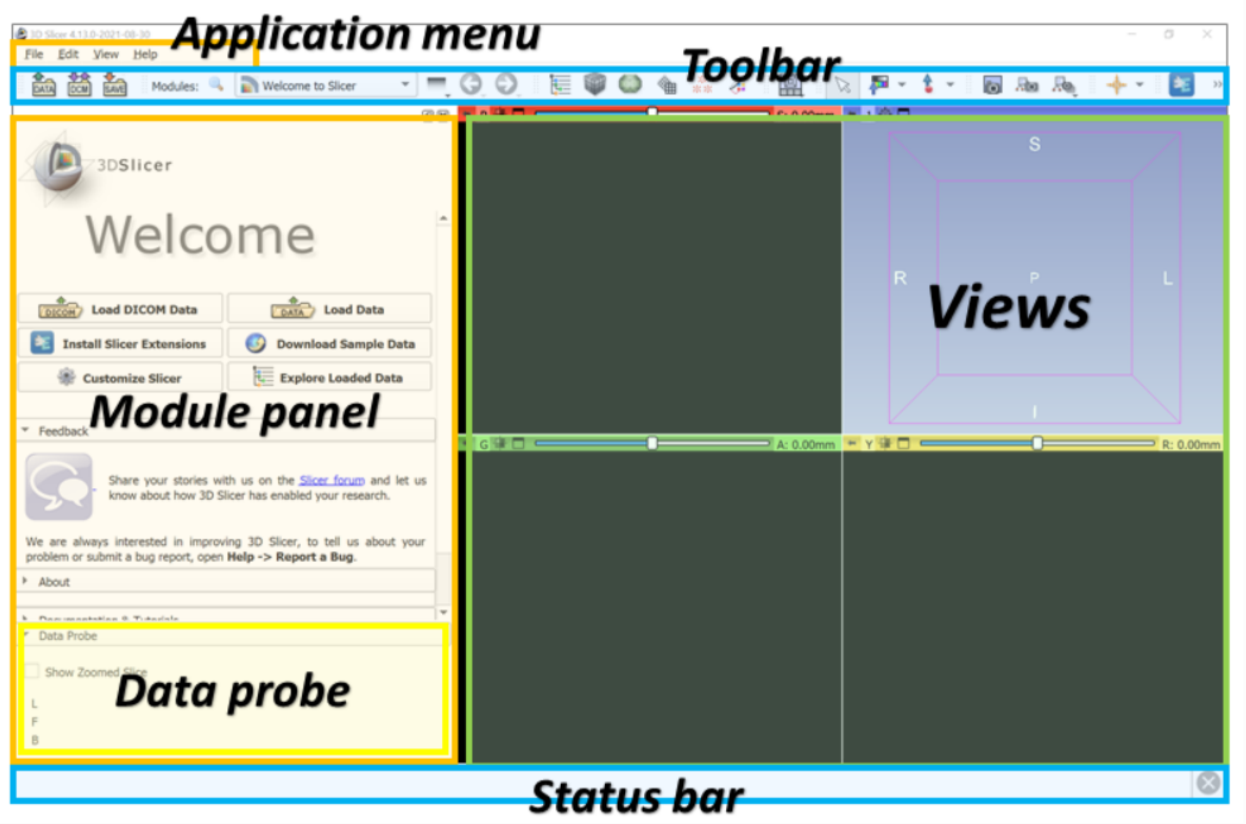

Protocol

1. If using the WACOM, in the folder containing the masks/images create a new folder. Rename with your name. Copy and paste the files into this folder, work on the files in the folder with your name.
2. Open Slicer
3. To load the CT data and the 3D model:

   File → Add data → Choose files to add [1] → hold ctrl and select “OSSO.nrrd” and “Segmentation.seg.nrrd” → Open [2] → OK

   [1] (Navigate to folder)

   [2] (in the add data into scene tab the two files you selected should appear with the box to the left checked and the description as Volume for the OSSO.nrrd file and Segmentation for the Segmentation.seg.nrrd file)

4. To visualize 3D model:

   Modules → Dropdown menu → Segmentations (1) → Module Panel Segmentations → Click circle in first column on the left (2)

S1 Fig 7: Loading segmentation masks into Slicer3D

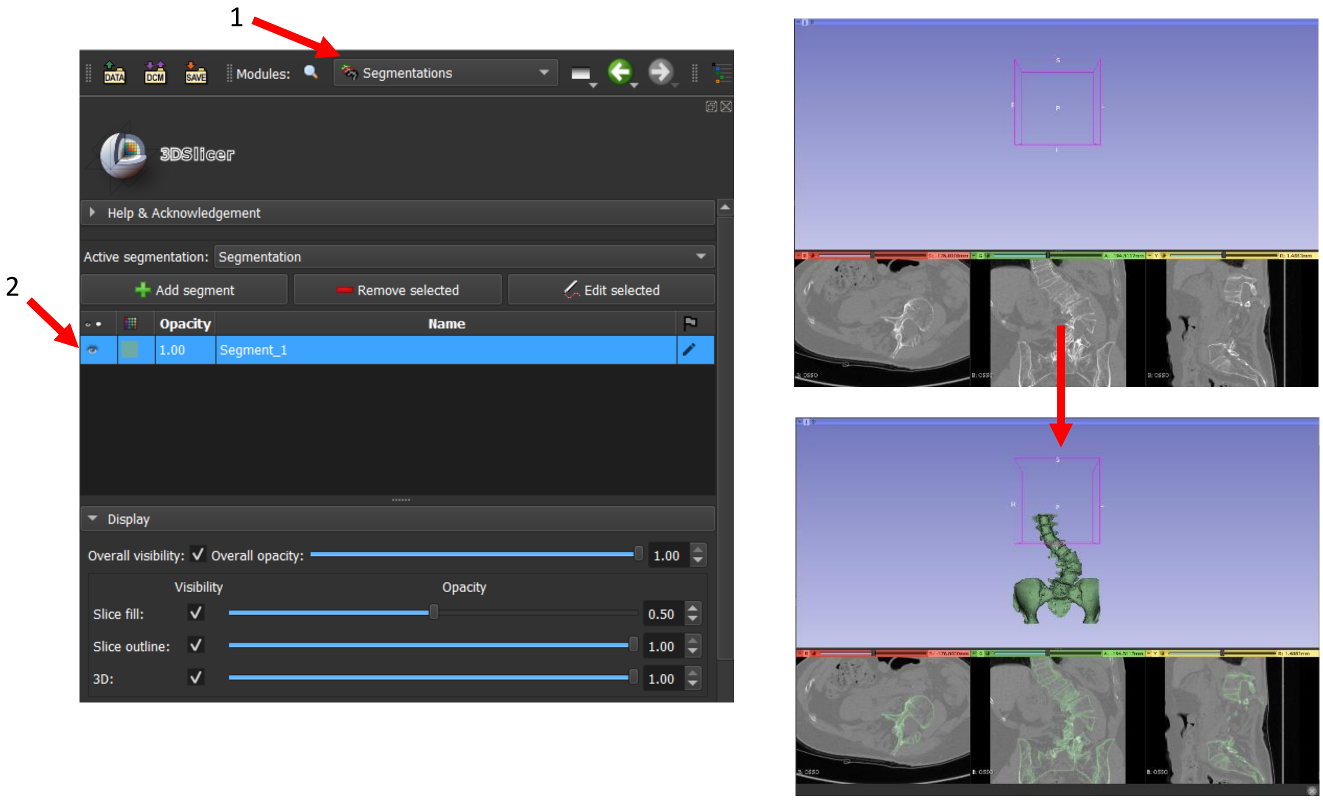

5. Marker settings and placing first marker:
   Place the first marker at the base of the sacrum (See Virtual Marker Palpitations Positions document, corresponds to marker S_1)
   Modules → Dropdown menu → Markups → Module Panel Markups → Create fiducial markups → Display [1] → Advanced → 3D Display → Placement mode: → snap to visible surface → Left click on model or slicer to place first marker

S1 Fig 8: Creating markers in Slicer3D.

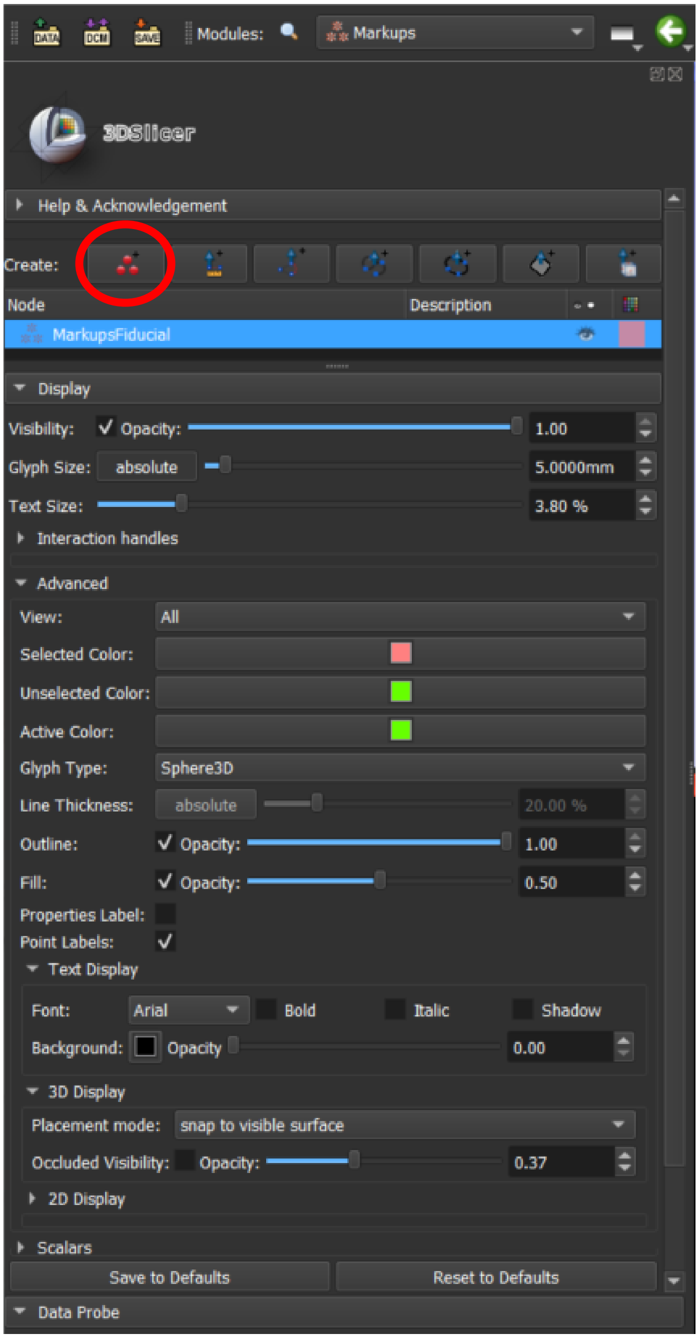

[1] Under display there are many other options, including colour, marker type ect, ones that may be helpful are Checking/Unchecking Point labels (to hide the marker name on the 3D view), Glyph Size which controls the size of the marker, and various Opacity options

6. Lock markup position - still in the Module Panel Markup:
   Control Points → Left click padlock to lock/unlock marker (if it has a red cross on the padlock the marker can be moved)

If marker is in the way hide it by clicking on the eye next to the padlock, after placing all the markers on a vertebra I recommend hiding all the markers on that vertebra.

S1 Fig 9: Locking the marker position.

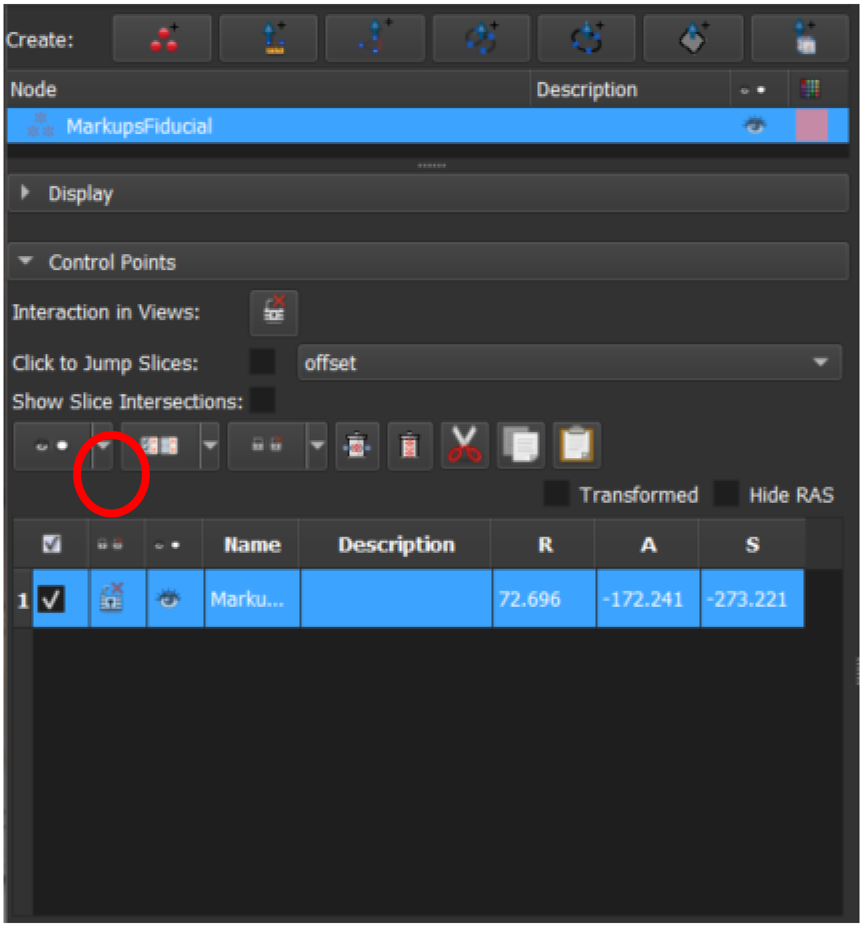

7. Name marker - still in the Module Panel Markup:
   Control Points → Under Name double left click on the text → Rename according to the Virtual Marker Palpitations Positions document.

8. For other markers - still in the Module Panel Markup:
   See Virtual Marker Palpitations Positions section for details of the order in which to place the markers
   Toolbar → Create and Place [1, 2] 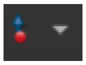 → Left click on model of slicer to place marker

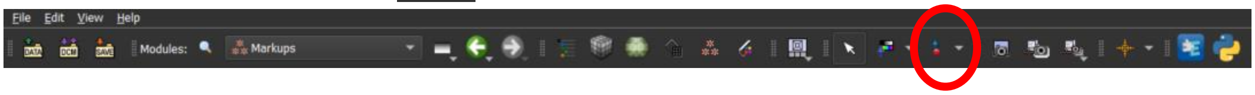

[1] If you cannot see the Create and Place button in the toolbar try View → Reset to Default
[2] In the dropdown of Create and Place make sure that Persistent is unchecked

9. Repeat steps 6 - 8 until all markers are placed
10. File → Save → Check box next to the fiducial marker set (will have name from when you created the fiducial markups), uncheck all other boxes, under file format ensure Markups Fiducial CSV (.fcsv) is selected → save
    It may throw a warning or an error, check that the file has saved (go to the directory where you saved it, check data and time of the file and that the last marker in the file corresponds to the last maker you placed) if these seem fine then ignore the error, otherwise contact me.
11. Close Slicer3D without saving

Tips and tricks:

- If you have locked a marker and later feel it is not correctly positioned, you can unlock it and move it and then relock it.
- You might find it helpful to isolate vertebrae or the sacrum, in which case make a copy of the segmentation

Toolbar → Python Interactor → Copy and paste code below Into the Python Interactor → press enter

```
segmentationNode = getNode("Segmentation")
sourceSegmentName = "Segment_1"

segmentation = segmentationNode.GetSegmentation()
sourceSegmentId = segmentation.GetSegmentIdBySegmentName(sourceSegmentName)
segmentation.CopySegmentFromSegmentation(segmentation, sourceSegmentId)
```

Rename the segment

Modules → Dropdown menu → Segment Editor → Module Panel Markups → Double left click on text below name and rename

Edit segment to isolate mask

Left click on copy of 3D segment → Select effect (I recommend Scissors) → Edit segment

Hide the segments that were not selected when editing as otherwise you will not see what you have done.

- Generally best to place the marker directly on the 3D model, and then check and fine tune its position on the slices. Placing the marker on 3D model may not always be possible in which case place it on the slices.
- View the CT slice on the 3D model (I've found axial and sagittal to be most helpful) can help with marker placement.
- In the case of a scoliotic spine the vertebrae are not aligned well with the planes of the CT scans so be careful when using them.
- Reducing the transparency of the 3d model can improve the marker placement Modules → Segmentation → Display → 3D → adjust slider
- Guideline for times, it takes about 30 minutes to place all the markers on a deformed vertebra, about 15-20 minutes on a healthy vertebra.

For mimics:

S1 Fig 10: Mimics interface.

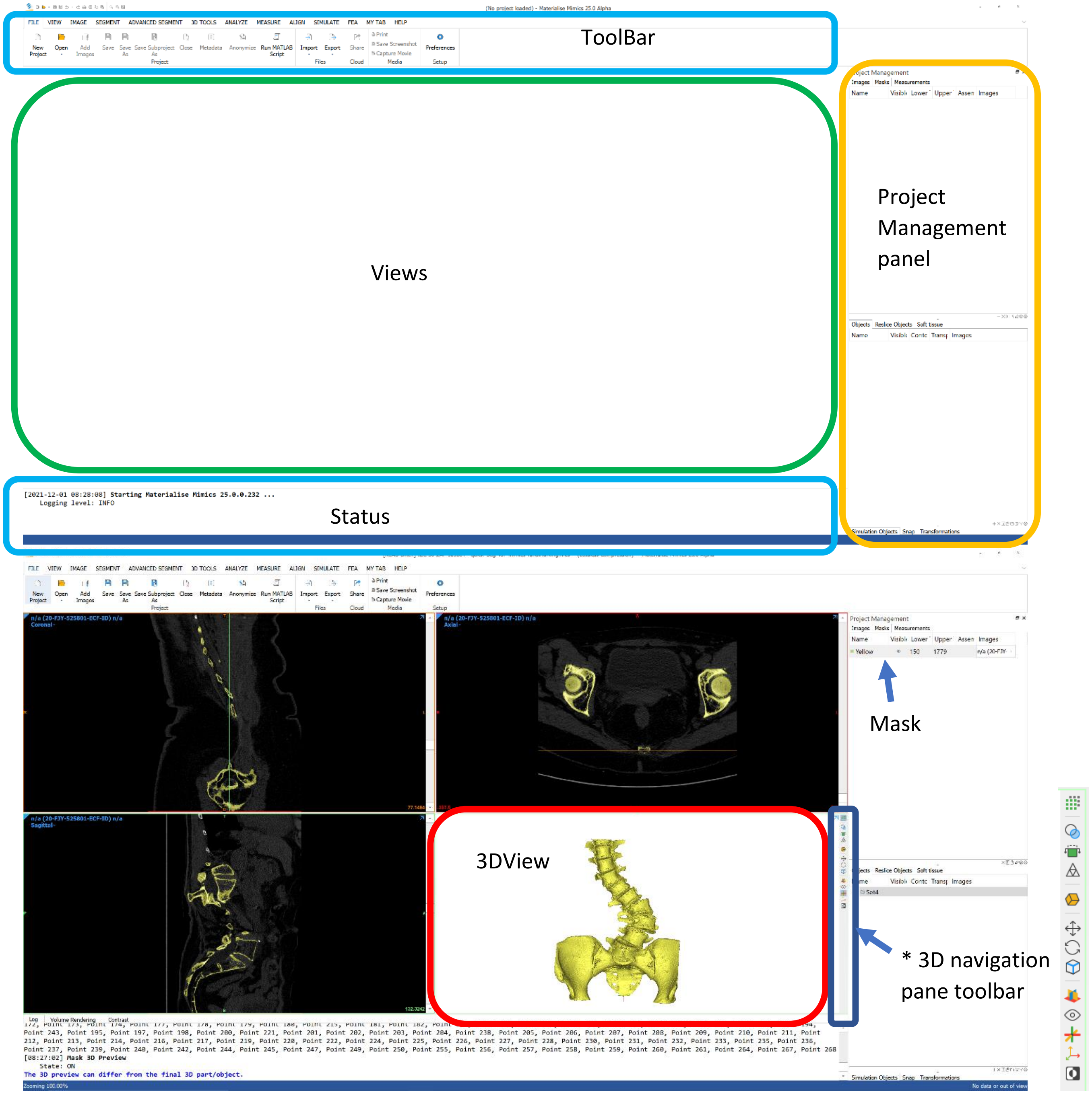

Protocol

1. If using the WACOM, in the folder containing the masks/images create a new folder. Rename with your name. Copy and paste the files into this folder, work on the files in the folder with your name.
2. Open mimics
3. Set up
   File → Open → Select quickSeg_for_mimics_landmarking.mcs → Open
4. Set up
   Project Management panel → Masks → Select Mask (named Yellow unless you change it)
5. Set up
   3D navigation pane → Tool bar* → Turn on Mask 3D Preview 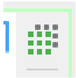
6. Set up
   3D navigation pane → Tool bar → Turn on 3D Navigation Indicator 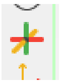
7. Placing of markers
   Left click on 3D mask preview at a marker position (see VirtualMarkerPalpitationsPositions), repeat until satisfied with position (check against all slice views and position on 3D mask) - first marker to place is at the base of the sacrum (See Virtual Marker Palpitations Positions document, corresponds to marker S_1)

S1 Fig 11: 3D visualisation of marker placement.

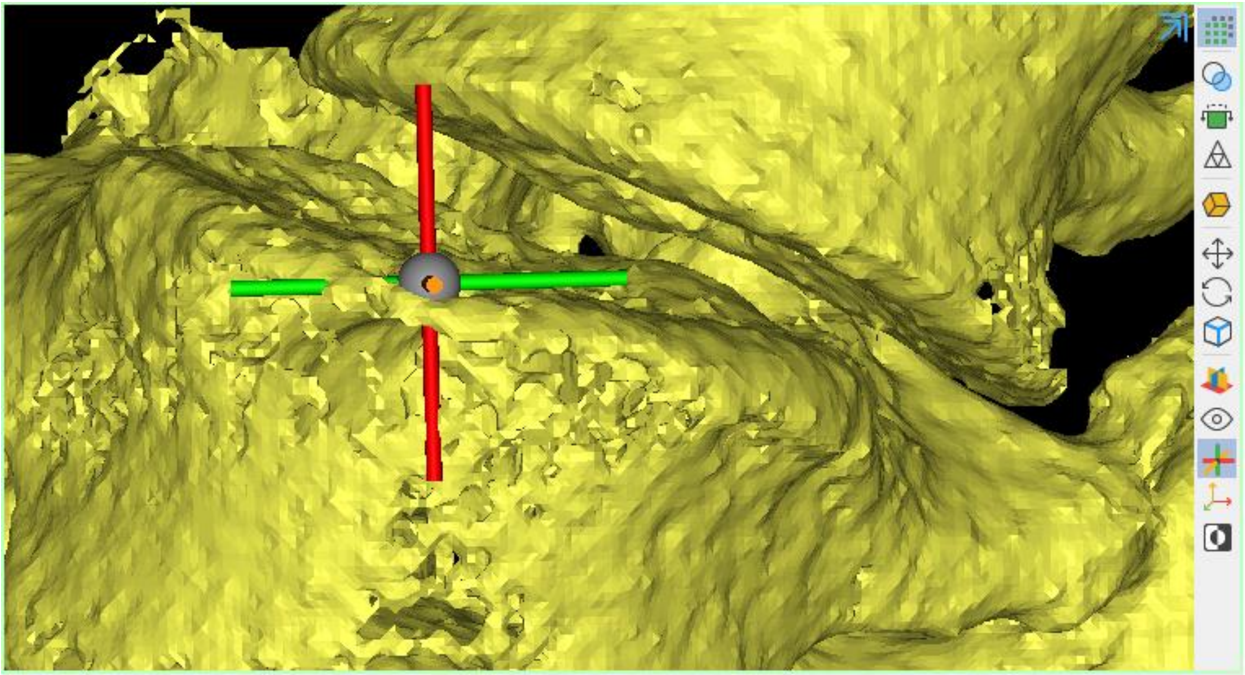

8. Placing of markers
   Toolbar → Analyze → Point → Left click in axial view at the intersection of the crosshairs to place marker → Esc

S1 Fig 12: 2D visualisation of marker placement.

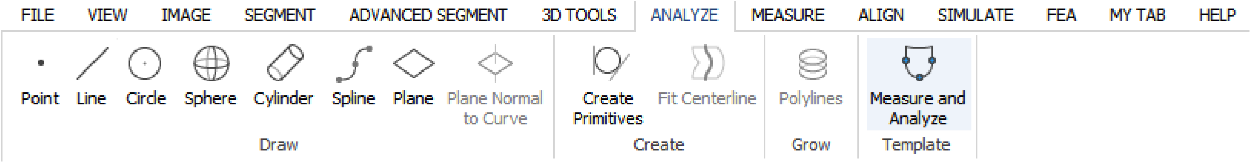

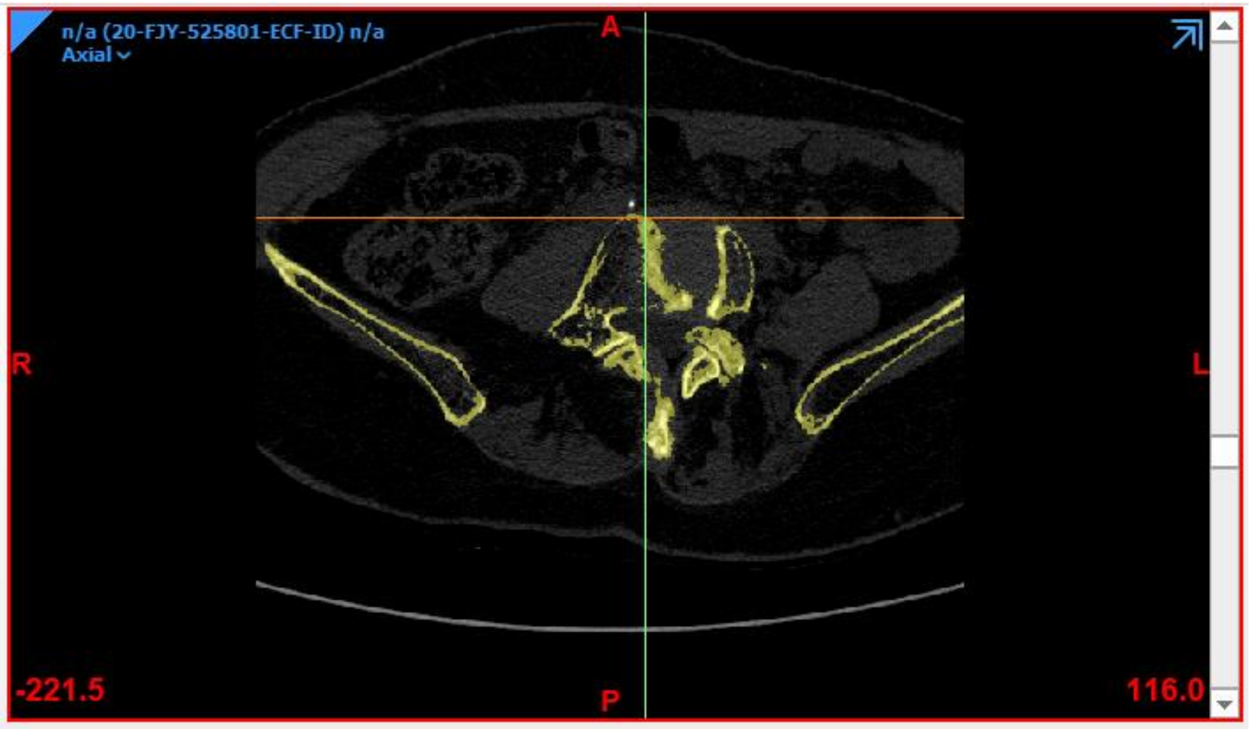

9. Placing of markers
   Project Management Panel → Objects (here you should see the point you just placed) → below "Name" double click on point you just created (named Point #) → according to the Virtual Marker Palpitations Positions document
   a. If marker is not helpful for the placement of other markers, Objects → Visible → left click on ellipse/eye to hide/unhide, this will avoid you accidently moving the marker

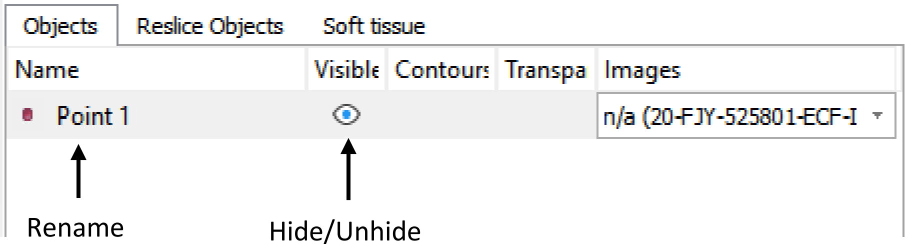

10. Placing of markers
    Repeat steps 6-8 to place all markers. See Virtual Marker Palpitations Positions document for details of the order in which to place the markers
11. Export markers
    Objects → ctrl a → right click on a marker label in the Objects tab → Export txt… → Add (check you know where file is being saved to, see path in output directory) → OK

S1 Fig 13: Exporting of markers.

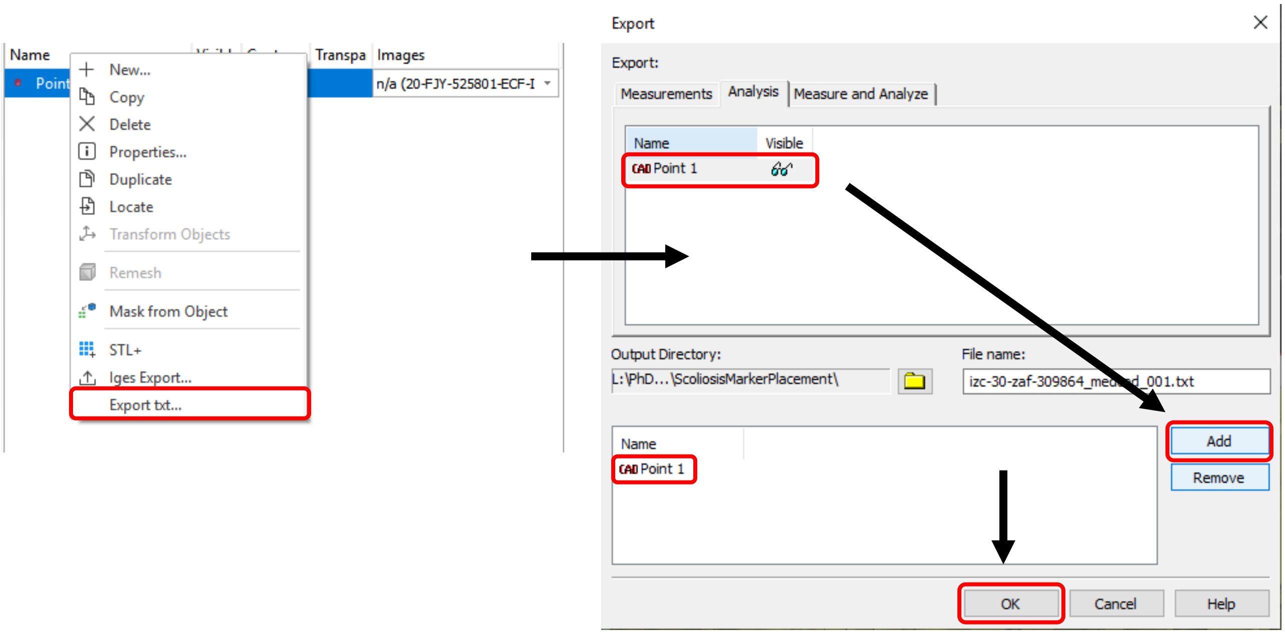

12. Close Mimics without saving project.

Tips and tricks:

You may find it helpful to isolate vertebra, in which case create a copy of the mask and then make the edits on there:

a. Project Management Panel → Masks → Right click on original mask → Duplicate Mask

b. Select mask you just created → Segment tab → Use tools of your choice, I recommend crop (draw box)
