# Supplementary material for: Generation of severely scoliotic subject-specific musculoskeletal models
Source: PLoS One. 2025 Dec 1;20(12):e0336211. doi: 10.1371/journal.pone.0336211 (PMC12668498; doi:10.1371/journal.pone.0336211)
Supplement: S2 File — Document describing in detail the codes that are provided as part of the workflow and how to use them. (PDF) [file pone.0336211.s002.pdf]

S2 Generation of scoliotic spine model:

Overview:

This document outlines the content of the download and how it was used and how future users can generate a scoliotic spine model.

Users are recommended to download and extract the zip folder – (.7z format is used, to extract use the 7-Zip tool https://www.7-zip.org/). The extracted folder can be moved to wherever the user wishes but the subfolder and file structure should remain the same (S2 Fig 1). Moving files or subfolders may result in the code failing as the paths to the files will be incorrect.

The material from this study (code, results, models, manual segmentations) can be downloaded from:

- Figshare: 10.6084/m9.figshare.28912175 – all the material ~ 1.25Gb.
- OpenSim: https://simtk.org/docman/?group_id=2618 – a lightweight download (~400Mb), contains all of the code and a minimal set of models and results that are needed to generate the rest of the results, and one example scoliotic spine model. The manual segmentations are not included and cannot be generated.

Structure overview of the download (after extracting from zip):

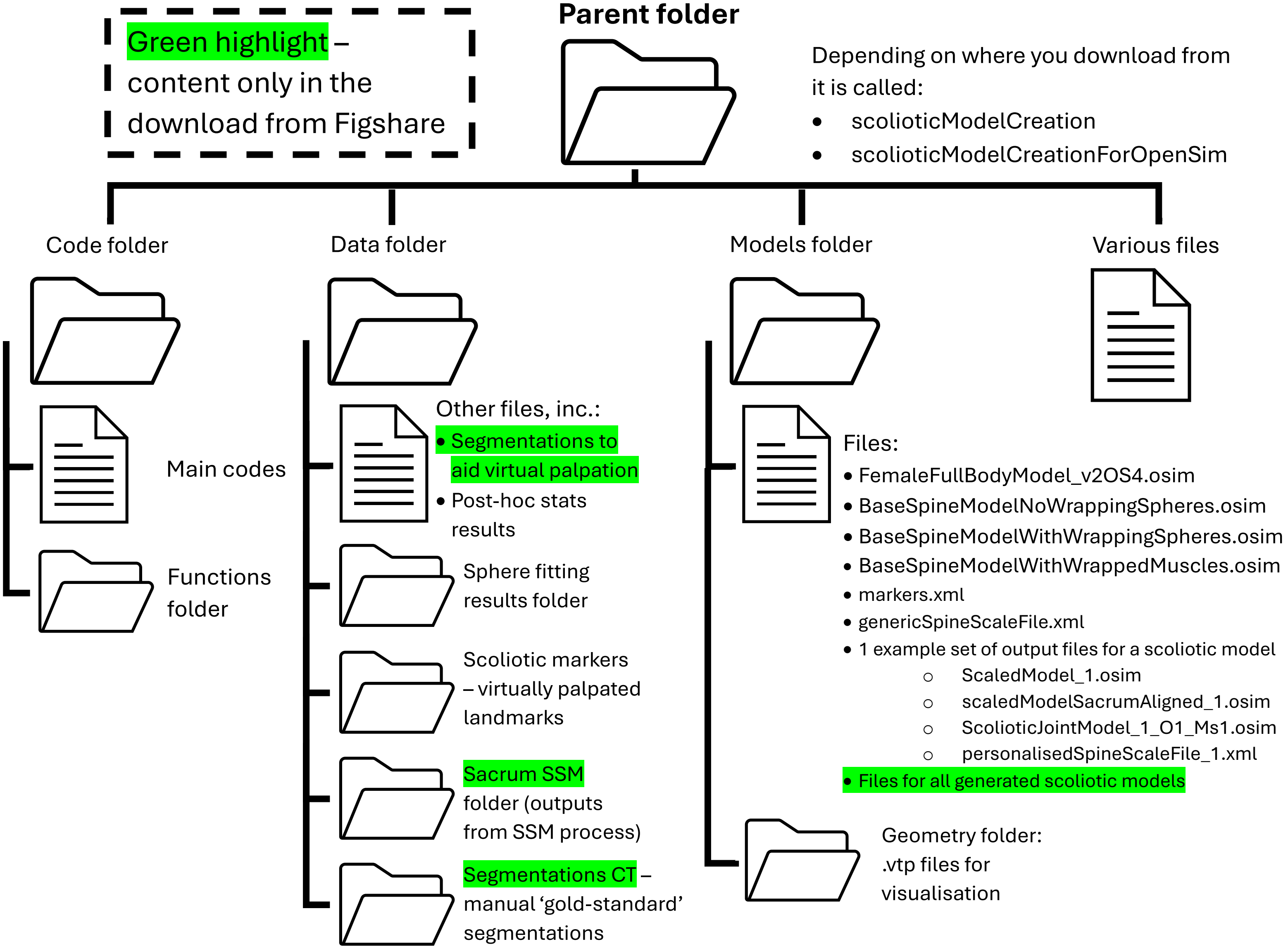

S2 Fig 1: Schematic of the folder structure of the download

Codes present in the download:

1. CreateBaseSpineModel.m – this simplifies the full body model from Bruno et al and adds the scaling markers, the ssm sacrum and the bushing forces (input model: FemaleFullBodyModel_v2, output model: BaseSpineModelNoWrappingSpheres)
2. DefineVertebra_sphere.m – this fits (position and size) a sphere to the vertebrae. These are used to define wrapping surfaces which are manually added to the opensim model. (inputs: .stl of the vertebrae. The model resulting from the manual addition of the wrapping spheres: BaseSpineModelWithWrappingSpheres)
3. WrapMuscles.m – this forces the muscles to wrap round the wrapping surfaces (input model: BaseSpineModelWithWrappingSpheres, output model: BaseSpineModelWithWrappedMuscles)
4. CreateScolioticSpine – Code to create scoliotic spine model (input model: BaseSpineModelWithWrappedMuscles, output model: ScolioticJointModel)
5. ModelVertebraCentres – Code to find the vertebral centres and fit a 4th order polynomial
6. markerVariability_IQR_Plot – code to plot the marker variability
7. JointMarkerAnalysis – analysis of the inter and intra-operator variability of the joint markers
8. scaleMarkerAnalysis – analysis of the inter and intra-operator variability of the scaling markers

Generation of a scoliotic spine model:

Users who wish to generate custom scoliotic spine models only need use the CreateScolioticSpine code.

Inputs for the CreateScolioticSpine code:

1. Required and provided:
    a. base model – BaseSpineModelWithWrappedMuscles.osim – provided
    b. genericSpineScaleFile.xml – provided
2. Required and user defined:
    a. set of virtually palpated anatomical landmarks according to the protocol defined in S1 Supporting Information - Virtual Palpation Protocol.
        i. For the code to find this file automatically it should be stored in the folder scolioticModelCreation/data/ScoliosisMarkers/operator#/set#. Users still need to specify the number (#), and the file should be named MarkerSet#. Alternatively the path can be manual specified in the code.
3. Optional:
    a. .stl files of the segmented vertebrae to allow for visualisation of the joint definition as the code runs.

Code description:

Here the order in which and how the code executes the operations to create the scoliotic spine model is described. Vertebrae L5-T1 and joints will be included in the model but only those specified will be edited to be subject specific. Variables within the code described here are coloured blue.

1. All paths and inputs are defined and necessary files are imported.
    a. The marker set provided by the user is stored in the subfolders operator#/set#. User should define the operator and marker set folder number. Ie. the user creates folder operator7/set5, and in this folder stores the file MarkerSet5.txt. In the code there is also an option to enter the full file path to the code This is specified by variables operator and markerSet.
    b. Operator specifies subject mass.
    c. Operator specifies the desired IVJ stiffness.
    d. The joints automatically included in the model are defined from S1 to T9.
        i. If the user wants other joints to be defined, then they need to specify them and the related child reference frames in the code in variable joints2Define and childReferenceFrames.
2. Marker set data is rotated to be in the reference frame of OpenSim and repositioned so that the sacrum apex is coincident with the OpenSim global origin.
    a. Rotations are applied by default a Z, Y, X rotation of 90, 90, 0. To change this change the rotations specified in the variable rotations. Change of units is also applied to variable markersCTinOSim.
    b. Markers are repositioned so that apex of the sacrum (label S_1) is coincident with the OpenSim global origin.
3. The model is scaled (by default only the vertebrae, manual edit of the code can allow the sacrum to be scaled).
    a. Scaling markers on the model and in the marker set are identified.
    b. Subject specific height (inferior-superior), depth (anterior-posterior) and width (left-right) of each vertebra and sacrum are calculated from the Euclidean distances between the positions of the virtually palpated anatomical landmarks.
    c. Process repeated using the markers on the base model.
    d. Scaling factor for each vertebra in each direction is then calculated (ratio of the model heights, depths, widths to subject specific heights, depths, widths).
    e. OpenSim scaling tool is used to scale the model – the generic scale tool provided with the code is edited with the scaling factors calculated and applied. Scaling programmatically caused the code to crash.
    f. Scaling file and scaled model is printed
4. Subject specific joint orientations are calculated:
    a. Using the S1_M3 and S1_M1 markers the sacral slope is calculated.
    b. Using the joint markers (labelled JM1-6) the joint orientations are calculated following the ISB recommendations.
5. Base model sacrum is aligned with the sacrum of the subject:
    a. Sacral slope of the base model is calculated (using the S1_M3 and S1_M1 markers).
    b. Rotations to move from the base model sacral slope to the subject specific sacral slope is calculated.
    c. Base model sacrum is rotated to align the sacral slope with the subject specific sacral slope.
    d. The base model sacrum is repositioned so that the apex of the sacrum (label S_1) is coincident with the OpenSim global reference (this has already been done for the virtually palpated marker set).
    e. Aligned model is printed out.

6. Scoliotic joints are defined, loop through each joint and:
    a. Get the model joint definition (origin and orientation) in the ground reference system.
    b. Move (rotations and translations) the base model joint definition to the subject specific joint definition in the global reference frame.
    c. Redefine the joint definition in the local reference frame of the parent body and apply to the model.
7. In the same loop as the joint definition, define the scoliotic vertebra alignment.
    a. Identify the endplate markers (labels M1-M8).
    b. Calculate the inferior-superior alignment (flexion-extension) of the vertebrae based on the centre of the endplates.
    c. Calculate the right-left bending alignment based of the vertebrae based on the lateral endplate markers.
    d. Reorientate the vertebrae.
8. Set the bushing forces to be coincident with the joints.
9. Print out the scoliotic model.
