# Supplementary material for: Generation of severely scoliotic subject-specific musculoskeletal models
Source: PLoS One. 2025 Dec 1;20(12):e0336211. doi: 10.1371/journal.pone.0336211 (PMC12668498; doi:10.1371/journal.pone.0336211)
Supplement: Attachment — Submitted filename: Response to Reviewers.docx [file pone.0336211.s004.docx]

**Reply to Reviewer #1**

**Reviewer #1:** The manuscript titled “Generation of severly scoliotic subject-specific musculoskeletal models” presents a semi-automatic workflow for the generation of subject-specific musculoskeletal models of the scoliotic spine, with a focus on severe deformities. The study is timely and relevant, and the effort to build a reproducible and open-source tool is truly commendable. The manuscript falls within the scope of Plos One journal and represents a relevant study in the advancement of musculoskeletal spinal biomechanics state-of-the-art. However, there are some points that require further clarification or improvement, below described:

**Authors:** The authors wish to thank the Reviewer for having read the paper, the positive comments, and the detailed recommendations for improving the paper.

**Reviewer #1:** The description of how the vertebrae were displaced relative to one another during the reconstruction process still requires improvements. The authors mention aligning the vertebrae based on virtually palpated anatomical landmarks and applying affine transformations, but the specific procedure used to reposition the vertebral bodies to achieve the target curvature lacks sufficient detail. Similarly, the method for anisotropic scaling of the vertebrae is not fully explained. Although affine transformations are said to be computed using specific landmarks, a more precise and step-by-step description would significantly improve the manuscript.

**Authors:** The relevant section in the methodology has been expanded to include these details (lines 268-319):

*“Following the virtual palpation, a scoliotic model was generated with the OpenSim libraries (18,45) (via a custom script). The workflow for this is as follows (S2 Supporting Information - Generation of scoliotic spine model):*

*1.	The reference systems of the model and the CT data (virtual palpation) were aligned by rotating the virtually palpated anatomical landmarks to correspond with the model reference system (Y axis in OpenSim corresponding to the inferior-superior direction of the CT data) and positioning the markers and the model such that the apex of the sacrum is coincident with the global OpenSim reference system.*

*2.	The model was scaled, specifically, the vertebrae and pelvis were scaled using affine transformations using the OpenSim scaling tool. Three scaling factors were calculated for each vertebra – corresponding to the anterior-posterior, inferior-superior, and right-left directions. The scaling factors were calculated as the ratio of the Euclidean distance between the existing landmarks on the model and the virtually palpated anatomical landmarks. Specifically, the anterior-posterior scaling factor was calculated using the mean position of the landmarks on the anterior- and posterior-most points of the inferior and superior endplates; the inferior-superior scaling factor was calculated using the mean position of the landmarks on the inferior endplate and the mean position of the markers on the superior endplate; the right-left scaling factor was calculated using the mean position of the landmarks on the right- and left-most points of the inferior and superior endplates. Their masses were scaled based on the total mass of the subject (subject-specific mass of 75kg was assumed). The sacrum was not scaled as an appropriate automatic landmark (apex of sacrum) could not be reliably virtually palpated on the CT scan due to the field of view.*

*3.	The sacrum of the model was aligned to the subject specific sacrum. The sacral slope was sacral slope defined in the CT data as the vector between virtually palpated anatomical landmarks on the posterior- and anterior-most points of the promontory of the sacrum.*

*4.	The joint poses of each IVJ were redefined following the ISB recommendation (57). For each joint this was implemented by first defining IVJ axes for the scoliotic subject. The right-left axis of the joint as the vector between the anatomical landmarks on the pedicles of the vertebrae and the joint origin (midpoint between the anatomical landmarks on the centre of the endplates immediately inferior and superior to the joint). Following this the anterior-posterior axis of the joint was defined as the average of the lines passing anteriorly through the anatomical landmarks on the midline of the endplates immediately inferior and superior to the joint and on a plane defined by the joint origin and the right-left axis. The inferior-superior axis was then defined as perpendicular to the right-left axis and anterior-posterior axis pointing cephalad. This defined the scoliotic joints in the global reference system of the model. The model joint definitions (origin and orientations) were then updated to correspond to the scoliotic joints.*

*5.	The vertebrae were realigned. Following the definition of each scoliotic IVJ, the vertebrae in the model were realigned based on the vertebral body alignment in the CT data. To define the alignment of the vertebrae a reference system for the body of each vertebra was defined in the global reference system. The inferior-superior axis of the vertebra was defined as the vector between the mean position of the landmarks on the inferior endplate and the mean position of the landmarks on the superior endplate; the anterior-posterior axis was defined as the mean of the vectors defined by the landmarks on the midline of the inferior and superior endplate; the right-left axis was defined as the cross product of the inferior-superior axis and the anterior-posterior axis. Using this definition the vertebrae were aligned in the corresponding parent joint reference frame.*

*6.	Finally, the bushing forces are realigned with the joint to avoid introducing preloads due to joint stiffness.”*

**Reviewer #1:** The definition of the intervertebral joint based on ISB recommendations is appropriate; however, the implementation itself would benefit from a brief explanation. Additionally, the manuscript mentions the addition of linear and spring dampers in the form of bushings, but several critical aspects are not clarified: were the connectors inserted after the morphological adjustments? Otherwise a pre-load would be introduced due to the roto-translation of adjacent vertebrae following the new curvature. Were the bushings replaced in the same relative location of traditional spherical joints?

**Authors:** The Authors have added a brief description of the IVJ definition according to the ISB recommendations (lines 239 - 245).

*“In brief, the axis defining right-left translation direction is defined by the direction of the line between the landmarks of the pedicles of the vertebrae; the axis defining the inferior-superior translation direction is defined by the lines that connect the landmark on the centre of the inferior and superior endplate of the vertebrae; the axis defining the anterior-posterior shear is then perpendicular to the other two axes; and the origin of the joint is the intersect of the lines passing through the landmark on the centre of the inferior and superior endplate of the two adjacent vertebrae.”*

The bushings are added before the morphological adjustments; the Authors would like to especially thank the Reviewer for noticing this detail in the methodology as they are absolutely correct. The code has been updated with the addition of a function that realigns the bushing forces with the joint reference frames after the morphological adjustments thus removing any preloads. This has been clarified in the code (lines 318- 319):

*“6.	Finally, the bushing forces are realigned with the joint to avoid introducing preloads due to joint stiffness.”*

Regarding bushing forces, it is important to note that these do not replace the joints rather they are as forces at the location of the joints, the authors hope this is clarified with the modifications to lines 182 - 186:

*“The original IVJs that allowed for the three rotational DoF were modified to also allow for the three translational DoF (for a total of 6 DoF at each IVJ). A lumped parameter spring-damper element (referred to in OpenSim as a bushing element) was then added at each IVJ, in the same relative location as the joints, to model its mechanical properties, as done by Christophy et al. (2012) (46).”*

**Reviewer #1:** Concerning the virtual palpation performed by the operators, it is unclear whether they were clinicians or engineers. This distinction is important, as it relates directly to the reliability of the process. Furthermore, it is essential to specify what instructions were given to the operators for the virtual palpation. Although the variability analyses are detailed and statistically thorough, without knowledge of the protocol followed by the operators, it is difficult to assess whether the observed reliability is due to the method itself or to the operators’ expertise.

**Authors:** The Authors have clarified that it was engineers that performed the virtual palpations following a protocol which has been added as supporting information (S1 Supporting Information - Virtual Palpation Protocol). The authors would like to stress that while a segmentation was used to aid the virtual palpation, this was not a detailed segmentation but one created by only applying a threshold, no further processing was done (lines 325-332):

*“To assess the inter-operator variability, the palpation was performed following a fixed protocol (S1 Supporting Information - Virtual Palpation Protocol) on the CT scan combined with a simple segmentation of the scan achieved with a single, quick thresholding operation with no further processing. The palpations were performed by five operators (all engineers, three of whom had extensive experience working with the spine in ex vivo, in vivo, and in silico studies), who repeated the procedure three times each (inter-operator dataset). Additionally, to evaluate the intra-operator variability, two of these operators (both with experience working with the spine) performed 10 repetitions (intra-operator dataset).”*

**Reviewer #1:** Model accuracy is primarily assessed based on vertebral center position and curve fitting. While this is a valuable metric, but how anatomically implausible results (e.g., muscle paths intersecting bone) were identified and addressed? Was this done purely via visual inspection, or was a systematic metric or validation method used?

**Authors:** There are two components of these models that can be assessed for geometrical and morphological accuracy of the models, firstly the accuracy of the bony structures ie. the position, size, and orientation of the vertebrae. These as the reviewer noted were assessed based on the vertebral centre position and curve fitting. The second component would be the geometrical and morphological accuracy of the soft tissues, in OpenSim models this corresponds only to the muscle paths (the cross-sectional area of a muscle would correspond to the muscle material properties). The accuracy of the muscle paths was only assessed with a visual inspection during the initial evaluation of the method (line 320). It was only used as an initial evaluation to confirm that the wrapping spheres placed on the vertebrae resulted in the intended behavior – ensuring that muscle-in-bone penetration could not happen. Secondly a more robust method to assess the muscle path accuracy was not used as a ground truth of the muscle paths was not available as the muscles were not defined well enough in the available CT data and no suitable MRI data was available to segment the muscles and functional test data was not available to perform indirect validations based on parameters (such as joint moments and contact forces) from simulation predictions. These details have been added to the limitations of the study (lines 693-698):

*“Furthermore, the accuracy of the muscle paths was only evaluated with a visual inspection in terms of anatomical plausibility (no muscle-in-bone penetration). Assessment of the patient-specific muscle path accuracy was not possible as magnetic resonance imaging (MRI) data was not available to establish the ground truth of the muscle paths. Indirect validation of the muscle paths based on the on parameters (such as joint moments and contact forces) from simulation predictions (77) was not possible as functional test data was not available.”*

**Reviewer #1:** A noticeable mismatch is observed between vertebrae L3 to L5 in Fig. 6. The lumbar vertebrae appear to be more spaced than expected, especially imagining the posterior region where the facet joints are located. It would be important to clarify whether this discrepancy is due to the scaling procedure, landmark definition, or reference alignment. Was this a visual artifact? How the authors think this could reflect a recurring issue in the model output?

**Authors:** The Authors believe this mismatch in the lumbar levels is most likely due to severely deformed L2 and L3 vertebra which can be seen in the gold standard manual segmentation. This makes virtual palpation of anatomical landmarks very challenging. Even when done well, the process for defining the IVJ (based on anatomical landmarks) assumes healthy vertebrae, as the geometry of these vertebrae is so different to that of a healthy vertebra, it calls into question the suitability of the IVJ definition for pathological vertebrae. The discussion has been expanded to address this (lines 628-636, 647-649):

*“There are multiple factors that may contribute to these systematic errors. The use of generic vertebra geometries rather than subject-specific geometries. The geometries can differ substantially, especially at levels where severe deformation is present, such as at L2 and L3 in the case of the subject used in the current study (Fig 2). This would reduce the correspondence of the positioning of the virtual palpitations between the model and the CT scan. This would alter the scaling and joint definition and would affect the position of the vertebral centres. One such example of this can be seen in the mismatch between vertebrae L2 to L5 (Fig 6) where the deformation of the L3 vertebra reduced the clarity of the L2 inferior endplate and the L4 superior endplate in the CT scan.”*

*“It should also be considered that the definition of the joint orientation was based on a non-pathological spine, meaning this definition may not be suitable for severe scoliosis.”*

**Reviewer #1:** In the results, lumbar lordosis is reported as a negative angle. While this is likely due to an inverted curvature, angle convention generally refer to positive angles. Moreover, in the table, some terminology doesn’t appear straightforwardly clear: what is intended for “main curve type” and “main secondary curve type” ? Do they refer to anatomical regions (e.g., thoracic vs. lumbar)? Similarly, the term “pelvic mismatch” is used without adequate context regarding its clinical significance or its impact on the model.

**Authors:** The Authors have updated the reported angles to respect the convention of using positive angles. The terminology refers to a commonly used clinical classification system – Lenke Classification. Reference to this has been added (line 263).

Additionally, the section in the discussion which addresses the spinopelvic parameters has been expanded to address the clinical relevance of these parameters (lines 680-689):
*“Spinopelvic parameters (such as pelvic incidence, pelvic tilt, thoracic kyphosis, and lumbar lordosis mismatch – the difference between the pelvic incidence and the lumbar lordosis (72)) and sagittal balance are important parameters in surgical planning and decision making and influence the outcome of surgical corrections (72–76). Poor management of these parameters has been associated with poor post-operative outcomes, such as post-operative lower back pain (72), proximal junctional kyphosis (73,74), distal junction kyphosis (74), disk degeneration (74), mechanical complications (instrumentation failure) (75), compensatory mechanisms to maintain balance (resulting in greater energy expenditure) (75), and generally lower health-related quality of life (76). Thus, evaluation of a wide range of spinopelvic parameters and spinal alignment is important.”*

**Reviewer #1:** The authors point out that the reconstructed models align well with CT scans acquired in the supine position but not with standing radiographs. This could turn into significant limitation of musculoskeletal models based on CT data. Since evaluations and model-based analyses are typically conducted in standing posture, the inability of the model to replicate anatomical parameters across postures could impact the reliability of the estimations. This limitation should be emphasized more strongly in the Discussion for further debate with scientific community.

**Authors:** The Authors have expanded the discussion to address this point (lines 663-671, 704-708):

*“The CT was acquired in the supine position, explaining the concordance between the model and the lumbar lordosis in the supine position. The standard deviation of the lumbar lordosis was even lower than that of the Cobb angle, suggesting the model was able to capture these two parameters accurately. This suggests that the workflow can create models that could be used for simulation of surgical procedures which are performed with the patient in the prone position. However, it presents a challenge for post-operative simulations when the subject may be upright and for simulations used to estimate the IVJ stiffness – which often use data from experiments that require the patient to be standing (70).”*

*“As part of this, methodologies will need to be found to move the model from a supine pose to an upright pose. One possible approach would be to perform a registration between the CT data (typically taken in a supine position) and a bi-planar X-ray (typically taken in a standing position) to calculate an individual transformation matrix for each vertebra to move between the two poses.”*

**Reviewer #1:** The statement on page 25 suggesting that systematic error affected the model more than operator variability appears in contradiction with earlier results where inter-operator differences were significant in several directions. Could the authors please motivate the sentence?

**Authors:** While there were statistically significant differences in the virtual palpation position of the anatomical landmarks (Table 4), this analysis did not consider the accuracy of the models, only the position of the anatomical landmarks. The statement regarding the systematic error was made in the context of the model accuracy, which was assessed with the median and the interquartile range of the errors of the generated models. In this study, as the only difference in the inputs to the models is the position of the virtually palpated landmarks, any variability in the model accuracy is accounted for by random error (ie operator variability of the virtual palpation). The median errors of the models represented systematic errors, this was typically larger than the interquartile range of the errors. The Authors have clarified this by expanding the results section (lines 532-539):

*“The median errors represent the systematic errors and the IQR errors represent the random errors which can be associated with operator variability. The overall IQR is 14% of the median RMSE in the Euclidean direction, the variability of the curvature accuracy is also 14-16% of the median error (Table 6). Analysing this based on the absolute error, the variability of the accuracy of the models (the IQR) (Table 7) is on average: 33% of the median absolute error in the anterior-posterior direction, 41% of the median absolute error in the inferior-superior direction, 35% of the median absolute error in the right left direction, and 22% of the median absolute Euclidean distance.”*

The Authors added a clarifying sentence to the section of the discussion on the operator variability (lines 594-595):

*“The analysis of the operator variability was based on the positions of the virtually palpated anatomical landmarks.”*

The Authors expanded the discussion on the model accuracy (lines 605-618):

*“Both systematic errors and random errors (due to operator variability in the positioning of the virtually palpated anatomical landmarks) affected the accuracy of the models. To assess the contributions of each, the median and IQR of the fitted curves (RMSE and R2) and the RMSE of the Euclidean distance of the centres of the vertebrae (Table 6) and the median and IQR of the absolute error in each direction were analysed (Table 7). The IQR of the RMSE of the vertebral centre and the RMSE and R2 of the curvature suggest that the overall model accuracy can be vary by around 15% due to operator variability, with larger variability (30-40%) seen when examining the absolute error in the individual directions. This implies the inter- and intra-operator variation affected the model accuracy less than the systematic errors. The difference between the errors due to the inter-operator variation and the errors due to intra-operator variation were negligible compared to the overall errors. Future work should focus on improving the accuracy of the model, this may focus on improving identification of the vertebra centre and the vertebral endplate centre. Furthermore, errors due to operator variability could be removed by automatically identifying the anatomical landmarks.”*

**Reviewer #1:** The issue of low reproducibility in vertebral geometry is mentioned, which is a known challenge in musculoskeletal modeling. In this perspective, does the proposed opensource tool requires a prior adjustment of vertebral geometry, or this is handled automatically by the workflow, or the method can also be applied to traditional vertebral .vtp files (which often suffer from low resolution)? The authors should consider adding a summary table or schematic—either in the Discussion or as Supplementary Material—outlining the expected inputs required for the tool shared opensource, which are the key steps, and the limitations of the current pipeline. This would greatly enhance the practical utility of the work.

**Authors:** The Authors have clarifies that for the sacrum a statistical shape model is used and for the vertebrae the original .vtp files are used (lines 205-208, 586-589).

The workflow only requires the users to provide the set of virtually palpated anatomical landmarks, therefore the Authors felt a full table or schematic was unnecessary. The discussion has been expanded to address this limitation. This has been clarified in the discussion (lines 579-593):

*“Users of the workflow only need to provide the set of virtually palpated anatomical landmarks (S1 Supporting Information - Virtual Palpation Protocol) and run the code to a create scoliotic spine models (S2 Supporting Information - Generation of scoliotic spine model). The code also allows for further modifications such as subject mass and subject specific IVJ stiffness. All the necessary codes and files are available on the OpenSim website (Personalised scoliotic spine models) and on FigShare (10.6084/m9.figshare.28912175). The workflow does not require the user to provide segmentations of the vertebrae, rather a statistical shape model of the sacrum and the original .vtp files of the vertebrae are used. The .vtp files of the vertebrae suffer from low resolution. Therefore, while the scaling may result in the model adequately represent the geometry of healthy vertebrae, in cases where the vertebrae are severely deformed the scaled model may not represent the deformed vertebra shape accurately. Furthermore, the code does not apply subject specific mass distribution or apply different stiffnesses at different IVJ levels. Future users could modify of the code to allow for these properties as well – a function is already provided to easily edit the IVJ stiffness, the desired stiffness simply needs to be specified.”*

Additionally, the Authors have prepared an additional Supporting Information document which fully describes how to use the code.

**Reply to Reviewer #2**

**Reviewer #2:** The introduction is largely clear and poses an interesting research aim. The promise of an open-source package to generate subject-specific MSK spine models from CT is very welcome.

Unfortunately, I believe that the manuscript lacks many relevant details and that the proposed code requires further development to achieve accuracy relevant for publication. Please see my more specific comments below:

**Authors:** The authors wish to thank the Reviewer for having read the paper and their detailed recommendations for improving the paper. Following the comments of both Reviewers, we modified the manuscript and we included substantial detail about the methods in general, and about the code. We believe we managed to significantly improve the manuscript thanks to the constructive criticism of the Reviewers.

**Reviewer #2:** 1. First, regarding the accuracy of the model, in the results a comparison is made with another paper where submillimeter accuracy was achieved, whereas the current proposed methodology leads to errors of multiple millimeters. Considering that this model was developed and tested on only one subject leads me to seriously doubt the reliability of the model. The authors rightfully mention in the discussion that 'a better definition or method is required to identify the vertebral endplate's center'.

**Authors:** The primary aim of the present study was to develop a codified, opensource workflow that enables a much more rapid generation of a patient-specific model by eliminating the need for time-consuming manual segmentation. This is a deliberate design choice to better support the practical implementation of the workflow in large-scale studies or time or cost-sensitive applications (such as surgical procedures). The workflow developed in the present study can produce a scoliotic model in a couple of hours. This is in contrast to gold-standard models which are more time-consuming as they require a manual segmentation of the entire spine – as the Authors report, segmentation of a single vertebra requires approximately an hour.

The Authors acknowledge that the time savings come with a modest reduction of accuracy compared to the gold-standard model in the referenced paper (which manually segments the entire spine – line 531). Nonetheless, the model remains within sub-centimeter accuracy (only a single order of magnitude greater than the sub-millimeter accuracy achieved by the gold standard). Future work should explore how the required accuracy varies across use cases. Furthermore, the reported accuracy likely represents a conservative estimate as the scoliotic case used to develop the workflow represents a complex case (a severely scoliotic case – Cobb 75°, with substantial apical vertebra deformation). We expect in a less complex case the model accuracy would increase, in part because identification of the vertebral endplate’s centre would be easier.

Unfortunately, as mentioned in the limitations, for this study only this single case was available. By publishing the workflow as opensource, we provide the wider scientific community with an accessible tool able to rapidly generate a scoliotic spine model, which can be validated, extended, and adapted for a wide range of cases and medical imaging data. This will establish the utility of the workflow for research and clinical use.

**Reviewer #2:** 2. A key point of the methodology is the virtual palpation of CT scans. The protocol for how to do this is missing. It is also not fully clear to me if the palpations are actually performed on the CT scans, or if they are performed on segmentations of the spine. If segmentation of the spine is required before palpations can be done, I do not believe there is much merit in this method over directly registering the vertebrae of the generic model to the segmentations.

**Authors:** The palpations are performed on the CT scans combined with a simple segmentation of the scan achieved with a single, quick thresholding operation with no further processing. While this includes a segmentation, it is drastically faster than the detailed segmentation of the spine that is necessary to register the vertebrae as the thresholding operation, is a single step process where the user only has to define the grey level to use as the threshold. This is now stated in the figure captions and has also been added to the body of the text now (lines 234-237 and 325-328):

*“A protocol defined two groups of virtual landmarks (S1 Supporting Information - Virtual Palpation Protocol), one to define the joint poses and the other to enable scaling (Fig 3), which are palpated on the CT scan combined with a simple segmentation of the scan achieved with a single, quick thresholding operation with no further processing.”*

*“To assess the inter-operator variability, the palpation was performed following a fixed protocol (S1 Supporting Information - Virtual Palpation Protocol) on the CT scan combined with a simple segmentation of the scan achieved with a single, quick thresholding operation with no further processing.”*

**Reviewer #2:** 3. In relation to the previous comment, it is unclear how the operators were instructed to perform the palpations and what their experience level is working with CT images (were these operators radiologists, orthopedic surgeons, students?).

**Authors:** The Authors have clarified that the palpations were performed by engineers and that they were given a protocol to follow. The protocol is also included as supporting information (S1 Supporting Information - Virtual Palpation Protocol). Lines 325-331:

*“To assess the inter-operator variability, the palpation was performed following a fixed protocol (S1 Supporting Information - Virtual Palpation Protocol) on the CT scan combined with a simple segmentation of the scan achieved with a single, quick thresholding operation with no further processing. The palpations were performed by five operators (all engineers, three of whom had extensive experience working with the spine in ex vivo, in vivo, and in silico studies), who repeated the procedure three times each (inter-operator dataset).”*

**Reviewer #2:** 4. A very big portion of the manuscript is about the interobserver and intraobserver variation. I believe this to be of minor relevance to the actual accuracy of the model. If instructions for virtual palpations are good enough to reach submillimeter repeatability, then that's sufficient. I suggest reporting the intraclass correlation coefficients and put more emphasis on the actual accuracy of the model. Especially the information about all repetitions (table 3) and comparisons between individual operators (table 4) doesn't mean much in my opinion.

**Authors:**

The authors have reported the intraclass correlation coefficients (lines 447-450). The authors decided to include the additional information regarding the inter- and intra-operator variability as the authors believe that this provides important context for the model accuracy, especially as the intent of the open-source codified workflow is for it to be re-used by other independent researchers. Furthermore, simplifying the analysis to the intraclass correlation coefficients would obscure the fact that despite excellent intraclass correlation, significant differences for specific marker positions were observed. The Authors believe this highlights the need to control for outliers when applying the protocol. This has been addressed in the discussion (lines 594-604).

The Authors have expanded the analysis of the model accuracy. The absolute errors of the Euclidean distance have been added (Table 7). Additionally, the Authors have expanded the analysis of the impact of the operator variability on the model accuracy. When considering the variability factor (inter or intra) the accuracy is similar. Therefore, the variability of the accuracy of the models should not be associated specifically with the inter- or intra-variability; nonetheless, the variability in the accuracy is due to the variability of the marker placement. This can lead to substantial variation in the model accuracy, the Authors have expanded the results section to address this (lines 505-508, 532-539):

*“the positions were generally consistent between models with limited variance, however there was an offset from the CT vertebral centres at many of the vertebral levels (Fig 6) indicating a component of systematic error in addition to the random error. This was consistent with the errors of the vertebral centres and polynomial curves (Table 6, Table 7).”*

*“The median errors represent the systematic errors and the IQR errors represent the random errors which can be associated with operator variability. The overall IQR is 14% of the median RMSE in the Euclidean direction, the variability of the curvature accuracy is also 14-16% of the median error (Table 6). Analysing this based on the absolute error, the variability of the accuracy of the models (the IQR) (Table 7) is on average: 33% of the median absolute error in the anterior-posterior direction, 41% of the median absolute error in the inferior-superior direction, 35% of the median absolute error in the right left direction, and 22% of the median absolute Euclidean distance.”*

The Authors have also refined the existing discussion and expanded the discussion to address this (lines 619-628):

*“As was the primary aim of the current study, the workflow allows for the rapid generation (15 to 30 minutes per vertebra to be included in the model) of a scoliotic MSK model. This was achieved by eliminating the need for the time-consuming process of manual segmentation which the current gold-standard models require (24), a process which the present study found requires approximately an hour per vertebra. However, the time-savings come with a modest reduction of accuracy compared to the gold-standard model. The gold-standard modelling approach from Overbergh et. al. 2020 (24) achieved mean errors of 0.71-1.59 mm depending on the direction and 2.09 mm in the Euclidian direction while the approach in the current study had median errors between 4.11-7.43 mm depending on direction and 11.32 mm in the Euclidian direction.”*

**Reviewer #2:** 5. The aim posed in the introduction strongly suggests that muscle-paths and IVJ stiffnesses are personalized. The IVJ stiffnesses are not personalized, and the muscle paths rely on the bone shape and predefined connection points of a generic healthy spine. The only true subject-specificity is the position and size of the vertebrae, the rest naturally follows from how the generic model was already developed and is thus not novel for this study.

**Authors:** The Authors have clarified the aim (lines 147-151):

*“develop an opensource package which provides the framework to generate scoliotic MSK models in OpenSim with subject-specific curvature, from CT data, while also automatically adapting muscle paths to follow the spinal curvature and allowing for the simple implementation of personalised IVJ stiffnesses;”*

The Authors would also like to draw attention to the methodology and discussion which states that the package includes a function to personalize the stiffness in any DoF (lines 190, 592, 703). Determining the personalized IVJ stiffness was not possible in this study as we were using data from a retrospective study and functional spine test data had not been collected. Methods for determining personalised IVJ stiffnesses have been the subject of previous studies, but a framework to easily implement of these stiffnesses into other models has not yet been provided – a custom code always needed to be written, or the model file had to be manually edited.

Regarding the muscle paths, this is not personalized (this was not possible from the available data). The novelty lies in the addition of wrapping surfaces to the vertebrae (which also scale with the vertebrae). This forces the muscles to path around the vertebrae compared to the original model which would allow the muscle paths to penetrate the bones. The Authors hope the re-wording of the aims is now clearer without delving too deeply into the methodological details.

**Reviewer #2:** 6. The effect of the palpations on the final positions of the vertebrae in the model is well-presented. It would also be interesting to see how this would affect actual modelling outcomes. Is the difference in position relevant for intended use of this model? The introduction mentions that these model can be used to predict treatment outcomes and simulate instrumentation strategies. It would be interesting to see some examples tested in this paper. Similarly, what is the effect of having the muscles go around the vertebrae on model predictions? And, what is the effect of adding the translational DoFs to the IVJ?

**Authors:** The Authors agree with the reviewer the impact of the palpations on the modelling outcomes would be very interesting and a study examining this, the effect of the muscles, and the presence of the translational DoFs is in fact a necessity before the workflow can be adopted into clinical practice. Simulations of surgical corrections to investigate these aspects were not included in the present study as it was outside the scope of the current study.

To investigate the effect of the palpations and therefore the vertebrae positions on surgical correction simulations two possible approaches to such simulations could be taken. One, a Monte Carlo simulation study however this would lack validation against a ground truth; alternatively an actual surgery could be simulated from which the appropriate motion and force data would need to be collected.

The effect of the muscles would likely be to apply additional forces on the vertebrae; the magnitude and direction of those forces would depend on the boundary conditions and the material properties assigned to the muscles. The Authors believe any work sufficient to draw meaningful conclusions would deserve a dedicated study.

The inclusion of the translational DoF better represents the physiological behavior of the IVJ. It will allow more accurate surgical simulations as relative translations of the vertebrae can be included, this is especially relevant in cases of scoliosis correction as height gain following the surgery is observed due to changes in intervertebral disc height – models which only include rotational DoF would be unable to explicitly model this.

The section on future studies has been expanded to reflect this (lines 709-714):

*“There are several key topics that should be the subject of future studies before models from this workflow is used in clinical scenarios, surgical simulations or for optimisation of implant design. The effect of the palpations (and thus the vertebra positions) on simulation outcomes and the effect of the muscle on the necessary surgical corrective forces should be investigated; additionally, methods to tune the muscle properties should be established to ensure physiological behaviour.”*

**Reviewer #2:** 7. The introduction states '...development of high-quality automatic segmentation methods, as the review by Galbusera et al. notes, work is ongoing (26).'. The reference here is 6 years old and plenty of development has been completed since then. Also already 5 years ago open-source neural networks have been published for automatic segmentation of the spine from CT, e.g.,:

Payer, C., Štern, D., Bischof, H., Urschler, M., 2020. Coarse to Fine Vertebrae Localization and Segmentation with SpatialConfiguration-Net and U-Net:, in: Proceedings of the 15th International Joint Conference on Computer Vision, Imaging and Computer Graphics Theory and Applications. Presented at the 15th International Conference on Computer Vision Theory and Applications, SCITEPRESS - Science and Technology Publications, Valletta, Malta, pp. 124–133. <https://doi.org/10.5220/0008975201240133>

**Authors:** The authors acknowledge the review mentioned is 6 years old and have added a number of more recent papers which demonstrate that while there has been development in the study of automatic segmentation the automatic segmentation of complex pathological cases (such as severe scoliosis) remains a challenge and continues to be a topic of ongoing study (lines 82-101):

*“Manual segmentation is a time-consuming process (22). The 2019 review by Galbusera et al. identified the application of artificial intelligence (AI) and machine learning for the development of high-quality automatic segmentation methods, noting that the performance of existing AI approaches could still improve substantially (26). The number of recent studies on the topic suggest that investigation into high quality automatic segmentation tools is ongoing (27–32). Two common approaches used in automatic segmentation are statistical shape modelling (32) and deep learning (27–31). However, both suffer with the drawback of requiring large clinically representative datasets to develop the automatic segmentation tools; the availability of such datasets is limited (especially of severely scoliotic cases) (27,33). Additionally, the training of these models is often associated with high computational memory requirements and computational cost (28).*

*The need for large clinically relevant datasets currently limits the applicability of such approaches in more complex cases such as vertebral fractures, scoliosis, and osteophytes (27,29,31,33). Complex geometries, such as vertebrae with osteophytes, have led to errors in segmentations (29), in particular for severe scoliosis the automatic segmentation has been suboptimal (30) compared to cases where the spinal alignment in the frontal plane is maintained (31). Furthermore, to the best to the author’s knowledge, such approaches for cases of severe scoliosis have not yet been incorporated into the larger pipelines that are required to create MSK models which would also require identification of attachment points, joint locations, and muscle paths.”*

**Reviewer #2:** 8. More details need to be mentioned regarding the patient and the scans under 2.1. Apparently there is also an x-ray that now only gets mentioned in table 2.

**Authors:** The authors have clarified in section 2.1 that the data available was CT data, X-ray data, and patient age and sex (line 174). Mention of the X-ray data has also been added to the overview of the methodology (line 164).

**Reviewer #2:** 9. For future reviews: please add line numbers to make referencing text easier.

**Authors:** The authors have added line numbers.
